# Projecting HIV Transmission in Japan

**DOI:** 10.1371/journal.pone.0043473

**Published:** 2012-08-20

**Authors:** Stuart Gilmour, Jinghua Li, Kenji Shibuya

**Affiliations:** Department of Global Health Policy, University of Tokyo, Tokyo, Japan; Lady Davis Institute for Medical Research, Canada

## Abstract

**Background:**

Little is known about the epidemiology of HIV in Japan, though newly-identified cases amongst men who have sex with men (MSM) show an increasing trend. Predictions of future trends in the HIV epidemic are essential to identify suitable interventions.

**Methods:**

A deterministic, compartmental model was developed that incorporated risk groups, disease stages, and treatment and testing parameters. This model was calibrated against current figures on new infections and run over 30 years to identify trends in prevalence amongst MSM, low-risk men and low-risk women. Multivariate sensitivity analysis was used to estimate sensitivity ranges for all outcomes.

**Results:**

Without new interventions amongst MSM in Japan, HIV prevalence will climb from its current rate of 2.1% to 10.4% (sensitivity range 7.4% to 18.7%), while HIV prevalence among low-risk men and women will likely decline. With small changes in safer sex behavior and testing rates, HIV prevalence can remain stable or even decline amongst MSM.

**Conclusions:**

Japan is at risk of an epidemic of HIV amongst MSM unless significant changes are made to its current public health intervention framework. More research is necessary to understand the key drivers of the epidemic in Japan.

## Introduction

Although HIV prevalence remains low in Japan [1], new HIV infections are at an all-time high, and have been increasing rapidly for the past 10 years. The majority of new infections in Japan are among men who have sex with men (MSM), and of these the vast majority are Japanese nationals [1]. This suggests a dynamic of increasing prevalence of the illness amongst a hard-to-reach, poorly-researched minority [1], [2], and the HIV epidemic among Japanese MSM may be following the same path it followed in MSM in other countries 20 years ago [3]–[5], with the same risk of reaching a high prevalence in Japanese MSM.

Recent research overseas has identified a promising role for biomedical interventions in preventing the transmission of HIV, and models in both developed [6] and developing [7] nation populations have shown the possibility that an intervention strategy based on universal access to voluntary testing and treatment (VCT) and antiretroviral treatment (ART) may contain or even eliminate the epidemic. Assessing how such strategies might work in Japan requires both an understanding of the current level of awareness and utilization of HIV testing in the Japanese population, and projections of the future path of the epidemic.

This paper presents projections of HIV transmission in Japan, employing a deterministic compartmental mathematical model which examines trends among MSM and non-MSM (male and female) over a 30 year period. The paper aims to explore the development of the epidemic under reasonable starting assumptions, to identify the key weaknesses in current research among MSM, to understand the extent of risk of an uncontrolled HIV epidemic among MSM, and what may need to be done to prevent the spread of this disease.

## Materials and Methods

### The Basic Model

A mathematical model of the epidemiology of HIV was developed to describe the progress of HIV through 10 compartments across three risk groups, based on an existing published model [6]. The 10 compartments represent stages of progression of HIV infection from HIV negative to AIDS, with HIV infection divided into symptomatic stages according to current treatment guidelines, and further divided into compartments according to level of serostatus knowledge. The model was developed using population parameters for the 15–59 age group in 2005, enabling comparability with current estimates of the size of the MSM population [8]. Prevalence figures from the Ministry of Health, Labour and Welfare [9] for the population aged 15–59 years of age. Figures for those infected through contaminated blood represented less than 3% of all PLWHA in 2011 [9] and were difficult to divide by sex and other risk categories, so these cases were excluded from the model.

The details of the model are provided in File S1: the compartmental structure is summarized in Figure S1, with accompanying symbols and their definitions in Table S1. Accompanying transmission modes are summarized in table S4. The model was solved numerically over a 30 year period, and key initial parameters were calibrated against data on new infections from 2006 to 2010. Based on US studies, it was assumed that 20% of all new infections remain unidentified [10], [11], and observed HIV infection numbers were adjusted accordingly before calibration. All baseline model parameters are summarized in Table S2, and starting populations are summarized in Table S3.

### Estimates of MSM

The number of MSM in Japan has been estimated at about 2% of the male population based on probability samples [8]. Although these probability surveys have low response rates and may not be representative, this proportion is consistent with estimates from other Asian countries. Because of the difficulty of identifying this population precisely, the population of MSM was included in sensitivity analysis.

### Risk Behavior

Information on number of partners and condom usage was obtained wherever possible from Japan-specific surveys of sexual behavior. These may not be representative population surveys, having been carried out through postal survey or through interview surveys in only a limited area [12]. All risk behavior variables were included in the sensitivity analysis in order to reflect this. No clear information was available on rates of partnership between MSM and women, so a nominal, small rate was assumed and given a wide possible range in sensitivity analysis.

### Prevalence Estimates

Counts of reported cases of HIV/AIDS by year until 2010 were obtained [9] and the combined count of men living with HIV/AIDS whose HIV was reported as due to “homosexual activity” or “unknown origin” was used to estimate the number of HIV cases among MSM. All other cases were considered to have occurred amongst the low-risk population. All observed values of the number of HIV cases were inflated by a factor of 2.7 to reflect the assumption that only 37% of non-AIDS HIV cases have been identified [13].

### Treatment and Screening Parameters

Treatment guidelines for Japan indicate that people with asymptomatic HIV and a CD4 cell count below 350 cells/mL should be advised to take up HAART in consultation with a doctor [14]. Because no research is available on treatment uptake rates amongst patients with asymptomatic HIV, treatment uptake was assumed to be 75% for patients in this stage, representing effective implementation of the guidelines. Rates of HIV testing are difficult to estimate in Japan, but some reports suggest that 13% of MSM in some community surveys received an HIV test in the past year [15]. Using data on total HIV tests conducted per year [16], [17], and assuming near-complete testing amongst pregnant women, it is possible to estimate the rate of testing amongst non-MSM. The population of people living with HIV was assigned to symptomatic or asymptomatic condition states in proportion to the inverse of the death rate for each condition (Table S3), which put about 75% of all people with HIV in the asymptomatic state. Passive case-finding was assumed to occur at twice the rate of the testing rate among non-MSM, since no research was available on the nature of case-finding procedures in Japan.

Very little information was available about rates of case finding, so these were assumed. Given that Japan has universal health coverage, it was assumed that 100% of symptomatic AIDS cases would be identified every year. At baseline, all AIDS cases were assumed to be identified and in treatment. Sizes of each cell of the compartmental model at baseline are given in Table S3.

### Modeling Two Scenarios

Two scenarios were modeled. The base case, modeled to represent the situation in Japan now, is referred to as the *Low HIV Awareness* scenario. A second counter-factual scenario, based on the assumption of successful but limited behavioral intervention, was also modeled. This is referred to as the *Higher HIV Awareness* scenario, and assumes higher rates of condom use amongst MSM, higher rates of HIV testing, and more effective passive case finding in people living with HIV/AIDS. The specific parameter values were:

- *Low HIV Awareness Scenario*: condom use amongst MSM and the low-risk population was 37% and 20% respectively, MSM were screened at a rate of 13% per year, and the passive case-finding rate was 10% per year
- *Higher HIV Awareness Scenario*: condom use amongst MSM and the low-risk population was 55% and 30% respectively, MSM were screened at a rate of 35% per year, and the passive case-finding rate was 40% per year

The values for the *Higher HIV Awareness* scenario represent a compromise between the current low values observed in Japan and the extremely high awareness of HIV in developed countries such as Australia, which has achieved testing rates of 50–70% and high condom use rates of about 60–80% [18].

### Multivariate Sensitivity Analysis

The range of possible values that could be used in the model and their effect on the overall outcomes was estimated using multivariate sensitivity analysis. This sensitivity analysis was implemented using Latin Hypercube Sampling (LHS), in which values were simultaneously sampled from specified distributions for several of the parameters considered most significant in the model. Under this method, the range of possible values for every parameter is divided into sections of equal width, and these sections are sampled without replacement. From within each section a value is sampled randomly, based on the assumed probability distribution, and the resulting set of values is then entered into the model. This enables the full range of possible values for each parameter to be sampled efficiently. The model results were compared to observed numbers of new HIV infections amongst males for a run-in period between 2006 to 2010, using the modeling efficiency parameter EF [19] to assess goodness of fit. One thousand Latin hypercube samples were taken, and the 200 models with the best EF statistic were retained for comparison with the baseline model. All models were then run for 30 years from 2010.

Table S5 summarizes values used in the multivariate sensitivity analysis. The triangular distribution was used to eliminate any risk of selecting negative values, while retaining the assumption that the value used for the point estimate was the most likely value.

## Results

### Baseline Scenario Projections

**Figure 1:**
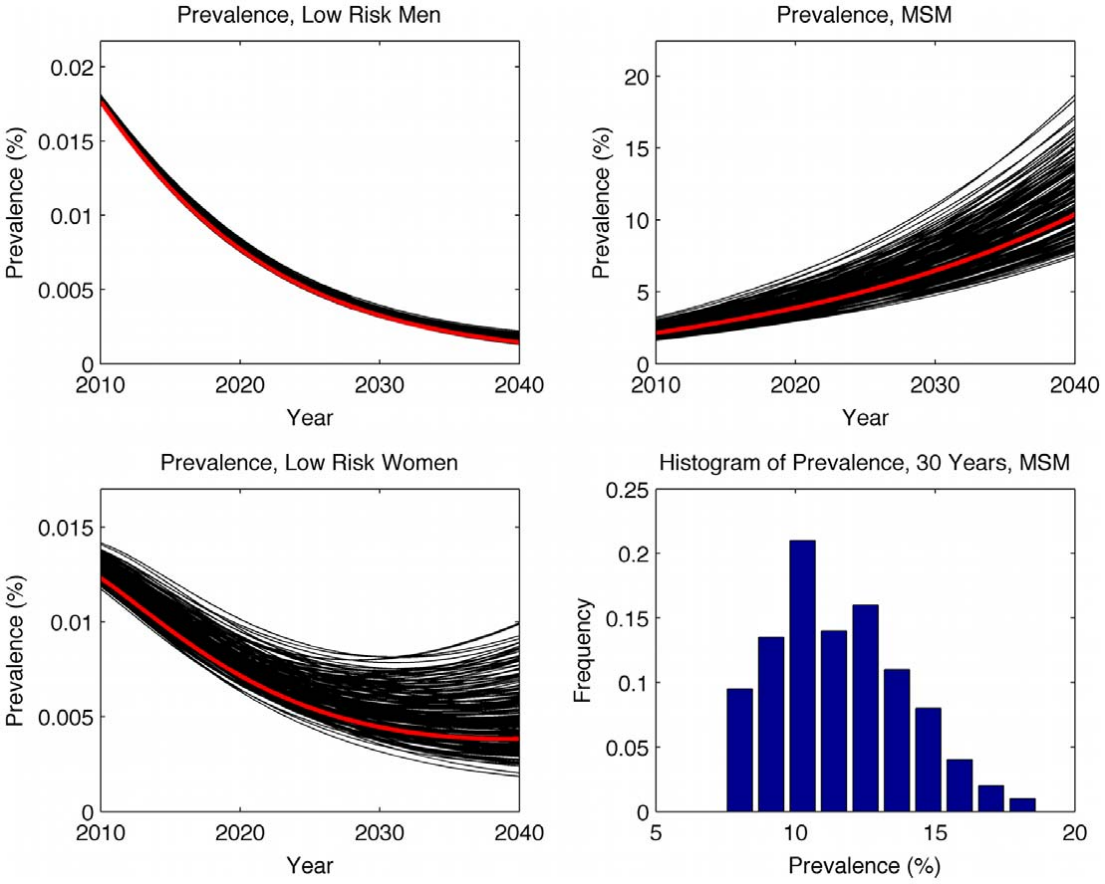
Prevalence estimates from the low HIV awareness scenario with Sensitivity ranges.

The model was run for 30 years under the baseline assumptions given for the *Low HIV Awareness* scenario, starting in 2010. The range of prevalence values for each of the three risk groups, and the histogram of HIV prevalence among MSM at year 30, is plotted in Figure 1. The values for the primary model are shown in red.

From a baseline value of 2.1%, prevalence among MSM increases over 30 years to 10.4% (sensitivity range: 7.4–18.7%). Prevalence decreases amongst low-risk men from 0.018% to 0.0014% (sensitivity range: 0.0013–0.0022%) and amongst women from 0.012% to 0.0038% (sensitivity range: 0.0019–0.0099%). The number of MSM was varied in sensitivity analysis to allow for uncertainty in the definition of this risk group, while counts of HIV cases remained fixed, with the consequence that initial prevalence rates entered into the model also varied, and it is clear from Figure 1 that the final prevalence over 30 years among MSM is highly dependent on the initial prevalence. This suggests that long-term prevalence amongst MSM in Japan is highly dependent on trends in prevalence over the next few years, and also implies that accurate knowledge of HIV prevalence in Japan is essential for predicting long-term trends of the epidemic.

For low-risk women the model bifurcates, and two model outcomes are possible. The lower range of values in Figure 1 corresponds with epidemic extinction, and shows HIV prevalence among low-risk women declining in a similar way to low-risk men. However, the higher range of values corresponds to epidemic growth, though still at a very low absolute level. Inspection of the relationship between parameter values and end-state prevalence in low-risk women suggests that the main driver of this bifurcation appears to be the rate of heterosexual contact with MSM.

### Alternative Scenario Projections

**Figure 2:**
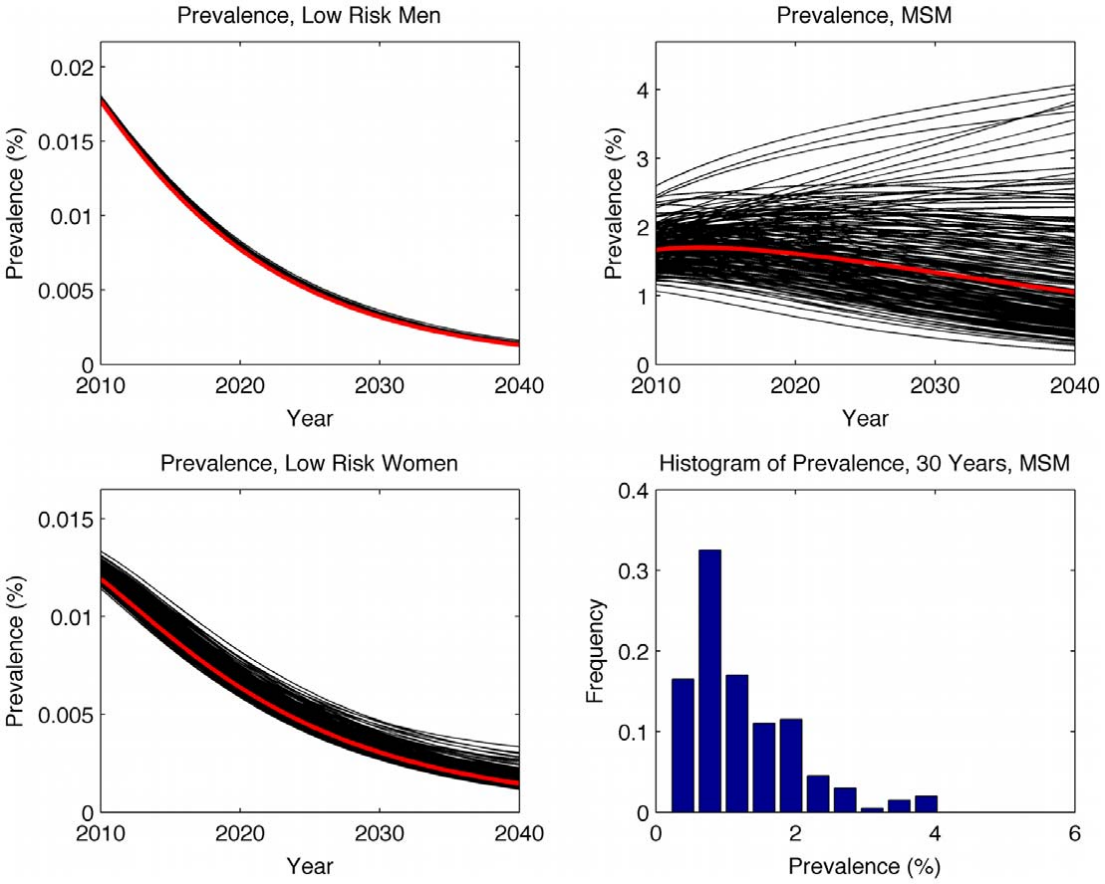
Prevalence estimates from the higher HIV awareness scenario with sensitivity ranges.

Prevalence estimates from the *Higher HIV Awareness* scenario, with sensitivity analysis ranges and a histogram of prevalence among MSM at year 30, are shown in Figure 2. The main model for this scenario is plotted in red. The prevalence at 30 years among MSM is 1.1%, with a sensitivity range between 0.2% and 4.1%. Among low risk men prevalence declines to 0.0013% (sensitivity range: 0.0012%–0.0016%) and among low risk women prevalence declines to 0.0015% (sensitivity range: 0.0012%–0.0033%). In the *Higher HIV Awareness* scenario the epidemic tends to extinction in both low-risk women and low-risk men, and appears to be slowly declining amongst MSM for the majority of cases. A small number of models show epidemic growth amongst MSM but this result does not appear likely in this sensitivity analysis. There is no evidence that heterosexual contacts between MSM and women are sufficiently infective as to produce epidemic growth amongst women.

The *Higher HIV Awareness* scenario differs from the *Low HIV Awareness* scenario only in that it models a small increase in condom use rates amongst MSM and a significant improvement in passive case-finding and voluntary testing rates amongst MSM. These changes would be sufficient to reduce the prevalence of HIV among MSM to approximately 1% over 30 years.

## Discussion

### Findings of the Models

Under the baseline assumptions given in Table 1, HIV prevalence amongst MSM will increase over the next 30 years from the current low level of about 2% to over 10%, while prevalence amongst the low-risk population will decline slowly over the same period. The epidemic will become increasingly entrenched among the MSM community, though there is evidence that the progress of the epidemic amongst low risk women depends strongly on the degree of overlap between the heterosexual and homosexual communities. Understanding the sexual risk patterns of bisexual and homosexual MSM in Japan and rates of sexual contact with women is thus essential to predicting the long-term patterns of HIV in women. The results of the *Higher HIV Awareness* scenario suggest that with consistent and realistic increases in rates of safe sex, limited intervention to improve voluntary testing rates amongst MSM, supported by better training and engagement of physicians who provide health services to MSM, the epidemic could remain stable and HIV prevalence could even decline over the long term.

### Limitations

Given the limited research available on MSM and HIV in Japan, in particular due to the lack of population-representative prevalence data [20], this model is clearly highly dependent on assumptions about key variables. The possible effect of these assumptions was tested using sensitivity analysis, and showed a wide range of possible outcomes. However, qualitatively, the majority of all possible outcomes from sensitivity analysis on this data indicate that the epidemic will continue to grow amongst MSM without rapid and sustained intervention. Furthermore, the goals of this intervention, in terms of improvements in condom use and voluntary testing rates, though high relative to current Japanese practice [15], are not unreasonable when compared to the behavior of MSM in other countries with mature but contained epidemics among MSM, such as Australia [18]. Sensitivity analysis of the Higher HIV Awareness scenario suggests that the epidemic can be contained in Japan if safer sex and testing behavior can be increased to lie within a range of achievable values, suggesting that although the specific parameters defining the future of the epidemic in Japan are not clearly understood, the broad findings of this study about the results of improved HIV awareness in MSM are robust.

### Implications for Intervention Planning

This study finds that HIV will prevalence will continue to increase unless sustainable changes in MSM risk behavior can be achieved. Current behavioral interventions amongst MSM in Japan are clearly insufficient, and more intensive, sustained and widespread interventions are necessary. Interventions should move from occasional community-specific events to an ongoing, organized campaign of awareness-raising and behavioral intervention. These programs should be consistent with international best practice interventions, which act simultaneously to improve MSM awareness of the risks of HIV infection, increase rates of testing, and establish a community context in which safer sex is considered the norm [21]. Such campaigns in other countries have required coordinated actions, including the establishment of specialist health centres, promotion of access to testing and treatment, legal changes, public health activism by gay rights organizations, intervention in sex venues and bars, and the widespread availability and promotion of condoms. Experience in these countries has shown that interventions of this kind can be implemented quickly and effectively at a community level where the political will exists [22]. Given the high rates of testing and treatment these campaigns require [7], [23], it is unlikely that biomedical interventions based on testing and treatment will be effective given current low rates of testing in Japan. HIV testing needs to be easily available, free and anonymous and all MSM should be encouraged to obtain testing regularly. Given that even extensive community, political and public health activism in places like Sydney and San Francisco has failed to see the elimination of this disease in MSM [24], [25], the continued fragmentary, low-level and unsustainable activities being conducted in Japan will be unlikely to have any significant impact on the spread of the disease. Japan’s public health community and MSM activists need to work together for a more open, forthright, active and sustained campaign to prevent HIV amongst MSM. Furthermore, Japan’s public health infrastructure readily supports a program to rapidly scale up testing in this community, by leveraging existing community organizations in areas with large numbers of MSM, and utilizing the existing network of public health centres (*hokensho*). Historical experience in developed nations indicates that such an expansion of testing and treatment is as much a question of political will and community awareness as availability of resources [26].

### Implications for Surveillance and Research

Available data on new cases of HIV suggests that the prevalence of HIV is increasing rapidly in men, especially MSM, and that the epidemic is effectively uncontrolled in MSM [1], [9]. However, very little information is available about the key parameters that drive the progress of HIV/AIDS in the Japanese population. Although efforts have been made to estimate the number of MSM, there appears to be very little information that can be used to identify key risk behavior.

Information about rates of testing and treatment in existing populations is essential not only for predicting the future progress of the HIV epidemic, but also for judging the potential effectiveness and cost-effectiveness of more refined interventions, such as pre- or post-exposure prophylaxis, the relative effectiveness of which depends heavily on the number of infections due to primary infection, and the rate at which new infections can be identified relative to existing infections [27]. Furthermore, guidelines for the effective implementation of pre-exposure prophylaxis depend on a detailed understanding of risk and testing behavior in the population [28] – information that is not readily available in Japan. Proper planning of Japan’s HIV prevention strategy thus depends heavily on better understanding of these factors.

With no evidence that HIV can be controlled under the assumptions given about the current knowledge and behavior of the MSM community, the future research agenda for HIV researchers in Japan needs to focus on well-designed, regular and widespread assessments of the sexual behavior of MSM, which requires:

1. Regular collection of data concerning number of regular and casual sexual partners; lifetime sexual behavior; condom use; overlap between MSM and heterosexual communities; HIV knowledge; and HIV testing behavior, through regular surveys similar to the Australian National Drug Strategy Household Survey in Australia [29].
2. Sentinel surveillance, similar to that conducted in Australia [30] that extends on the current notification system [31] to enable identification of the sexual identity of those receiving HIV testing and the reasons for this testing, including attempts to estimate the proportion of new infections detected every year. Incidence databases established amongst doctors providing services to MSM, and/or at health clinics providing anonymous testing, would enable a better understanding of incidence rates and levels of detection of new infection, as well as monitoring disease progression, reducing overlap of case notifications, and enable more accurate assessment of the duration of unidentified asymptomatic infection in cohorts of PLWHA.
3. Rapid assessment surveys amongst at-risk groups – particularly MSM – including prevalence testing by finger-prick sampling, and basic risk behavior, similar to those implemented at Needle/Syringe Programs in Australia [32].
4. Research amongst doctors who work with MSM, for sentinel surveillance and understanding MSM’s voluntary testing and ART treatment behavior.
5. Establishment of a clinical cohort of PLWHA, in order to better understand disease progression and survival rates in the Japanese context.

### Conclusion

The HIV epidemic at its current stage in Japan is vulnerable to even small changes in sexual behavior, and could potentially be brought under control – even amongst MSM – within a generation if these small behavioral changes, and improvements in active and passive case-finding, were to occur soon. However, there is a significant risk that the epidemic will grow out of control in the near future. HIV researchers need to focus on identifying the key behavioral factors driving the epidemic, to facilitate change in these behaviors and ensure that the disease does not get a solid foothold in Japan.

## Supporting Information

Figure S1 **HIV transmission compartmental model structure.**
(TIF)

Table S1 **Summary and Description of Model Variables.**
(DOCX)

Table S2 **Baseline model parameters.**
(DOCX)

Table S3 **Initial values for the populations of risk groups.**
(DOCX)

Table S4 **Modes of HIV transmission between risk groups.**
(DOCX)

Table S5 **Parameters used in sensitivity analysis.**
(DOCX)

File S1 **Mathematical Model and Equations.**
(DOCX)
