# Supplementary figures and images for: Projecting HIV Transmission in Japan
Source: PLoS One. 2012 Aug 20;7(8):e43473. doi: 10.1371/journal.pone.0043473 (PMC3423344; doi:10.1371/journal.pone.0043473)

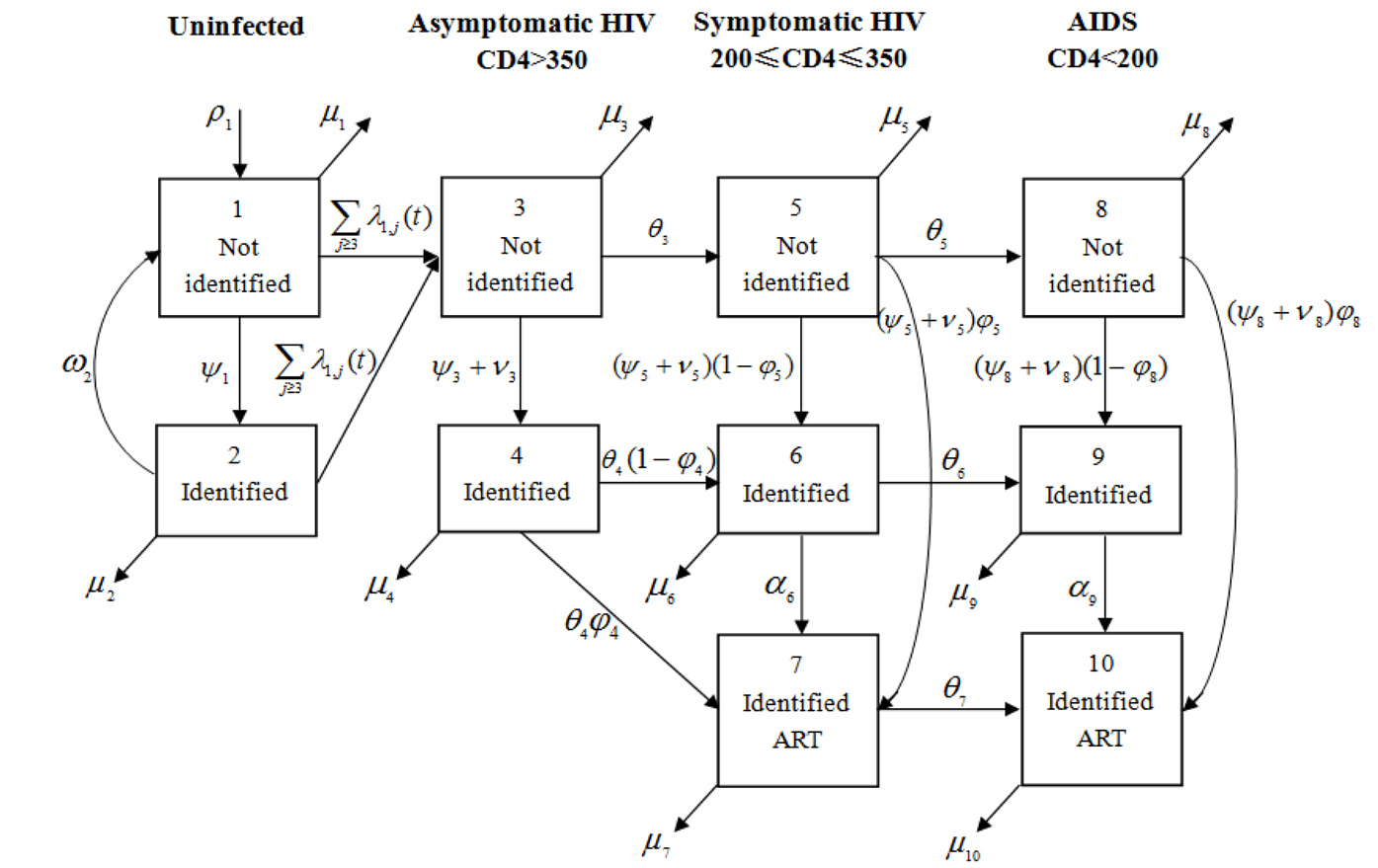

Supplement: Figure S1 — HIV transmission compartmental model structure. (TIF) [file pone.0043473.s001.tif]
