# Supplementary material for: Projecting HIV Transmission in Japan
Source: PLoS One. 2012 Aug 20;7(8):e43473. doi: 10.1371/journal.pone.0043473 (PMC3423344; doi:10.1371/journal.pone.0043473)
Supplement: Table S1 — Summary and Description of Model Variables. (DOCX) [file pone.0043473.s002.docx]

| **Variables/Symbols** | **Definition** |
| --- | --- |
| **Demographic characteristics** | |
| $X_{i,j}$ | Number of people in risk group *i* with status *j* |
| $b_{j}^{i}$ | Annual background mortality rate |
| $\mu_{j}^{i}$ | Annual mortality rate due to HIV/AIDS |
| $\mu_{1}^{i},\mu_{2}^{i}$ | Annual maturation rate |
| $\rho_{1}^{i}$ | Annual entry rate |
| **Sexual transmission** | |
| $\sigma_{f,m}^{Z}$ | Annual transmission probability per partnership from female to male, where z= asymptomatic HIV, symptomatic HIV, and AIDS |
| $\sigma_{m,f}^{Z}$ | Annual transmission probability per partnership from male to female, where z= asymptomatic HIV, symptomatic HIV, and AIDS |
| $\sigma_{m,m}^{Z}$ | Annual transmission probability per partnership from male to male, where z= asymptomatic HIV, symptomatic HIV, and AIDS |
| $n_{1}^{S}$ | Annual same-sex partners of MSM |
| $u_{1}^{S}$ | Condom use with same-sex partners, percent |
| $n_{i}^{O}$ | Annual opposite-sex partners in risk group *i* |
| $u_{i}^{O}$ | Condom use with opposite-sex partners in risk group *i* |
| $\kappa$ | Condom effectiveness |
| **HIV screening** | |
| $\psi_{j}^{i}$ | Fraction of population tested in past 12 months for risk group *i* with status *j*, percent |
| $1/\omega_{2}^{i}$ | Average duration (years) that uninfected individuals remain identified after screening in risk group *i* |
| $\nu_{j}^{i}$ | Annual probability of symptom-based case finding in risk group *i* with status *j*, percent |
| $r_{1}$ | Reduction in sexual behavior among persons identified as HIV-positive, percent |
| $r_{1}^{'}$ | Reduction in sexual behavior among AIDS patients, percent |
| **ART Treatment** | |
| $\phi_{j}^{i}$ | Fraction starting ART at CD4 cell count of 350 in risk group *i* with status *j* |
| $\alpha_{j}^{i}$ | Annual ART entry rate if CD4 cell count <350 of risk group *i* with status *j* |
| $r_{2}$ | Reduction in sexual infectivity due to ART, percent |
| **Others** | |
| $\theta_{j}^{i}$ | HIV disease progression rate for individuals in risk group *i* with status *j* |
| $\sum_{j\geq3} \lambda_{i,j}(t)$ | Transmission forces for each risk groups *i* |
