# Supplementary material for: Projecting HIV Transmission in Japan
Source: PLoS One. 2012 Aug 20;7(8):e43473. doi: 10.1371/journal.pone.0043473 (PMC3423344; doi:10.1371/journal.pone.0043473)
Supplement: Table S2 — Baseline model parameters. (DOCX) [file pone.0043473.s003.docx]

| **Variable** | **Value** | **References** |
| --- | --- | --- |
| **Demographic characteristics** |  |  |
| Annual mortality rate (background) |  |  |
| Men | 0.0045 | ([1](#_ENREF_1)) |
| Women | 0.0092 | ([1](#_ENREF_1)) |
| Annual mortality rate (due to HIV/AIDS) |  |  |
| Asymptomatic (CD4>350) | 0.02 | ([2](#_ENREF_2)) |
| Symptomatic (200<CD4≦350) | 0.063 | ([2](#_ENREF_2)) |
| AIDS (CD4≦200) | 0.22 | ([3](#_ENREF_3), [4](#_ENREF_4)) |
| Annual mortality rate (due to HIV/AIDS) |  |  |
| Symptomatic with ART | 0.05 | ([2](#_ENREF_2)) |
| AIDS with ART | 0.075 | ([2](#_ENREF_2)) |
| Annual maturation rate |  |  |
| Men | 0.0271 | Calculated, Census Data |
| Women | 0.0232 | Calculated, Census Data |
| Annual entry rate |  |  |
| Men | 0.0167 | Calculated, Census Data |
| Women | 0.0162 | Calculated, Census Data |
| Initial population (aged 15-59) |  |  |
| MSM | 680,000 | ([5](#_ENREF_5)) |
| Low-risk population |  |  |
| Men | 38,056,434 | Calculated, Census Data |
| Women | 37,491,351 | Calculated, Census Data |
| Initial prevalence (aged 15-59), % |  |  |
| MSM | 2.1% (1.2-4.7%) | Calculated: ([6](#_ENREF_6)),([5](#_ENREF_5)) |
| Low-risk population |  |  |
| Men | 0.03% | Calculated: ([6](#_ENREF_6)), Census |
| Women | 0.02% | Calculated: ([6](#_ENREF_6)), Census |
| **Sexual transmission** |  |  |
| Transmission probability per partnership |  |  |
| Heterosexual (female to male) |  |  |
| Asymptomatic HIV | 0.01 | ([7](#_ENREF_7)) |
| Symptomatic HIV | 0.02 | ([7](#_ENREF_7)) |
| AIDS | 0.03 | ([7](#_ENREF_7)) |
| Heterosexual (male to female) |  |  |
| Asymptomatic HIV | 0.03 | ([7](#_ENREF_7)) |
| Symptomatic HIV | 0.04 | ([7](#_ENREF_7)) |
| AIDS | 0.08 | ([7](#_ENREF_7)) |
| Homosexual (male to male) |  |  |
| Asymptomatic HIV | 0.04 | ([8](#_ENREF_8)) |
| Symptomatic HIV | 0.05 | ([8](#_ENREF_8)) |
| AIDS | 0.12 | ([8](#_ENREF_8)) |
| Annual same-sex partners |  |  |
| MSM | 5.5 | ([9](#_ENREF_9)) |
| Annual opposite-sex partners |  |  |
| MSM | 0.1 | Assumed |
| Low-risk population |  |  |
| Men | 1.1 | Calculated: ([10](#_ENREF_10)) |
| Women | 1.12 | Calculated^a^ |
| Condom use with same-sex partners, % |  |  |
| MSM | 37% | ([9](#_ENREF_9), [11](#_ENREF_11), [12](#_ENREF_12)) |
| Condom use with opposite-sex partners, % |  |  |
| MSM | 20% | ([13](#_ENREF_13)) |
| General population |  |  |
| Men | 20% | ([10](#_ENREF_10), [13](#_ENREF_13)) |
| Women | 20% | ([10](#_ENREF_10), [13](#_ENREF_13)) |
| Condom effectiveness | 0.9 | ([14](#_ENREF_14)) |
| **HIV screening** |  |  |
| Proportion of population tested in past 12 months, % |  |  |
| MSM | 13% | ([12](#_ENREF_12)) |
| Low-risk groups | 5% | Calculated:([15](#_ENREF_15), [16](#_ENREF_16)) |
| Average period that uninfected individuals remain identified after screening, years | 1 |  |
| Annual probability of symptom-based case finding, % |  |  |
| HIV | 10% | Assumed |
| AIDS | 100% | Assumed |
| Reduction in sexual partners among identified HIV-positive, % | 20% | ([17](#_ENREF_17)) |
| Reduction in sexual behavior among AIDS patients, % | 90% | Assumed |
| **ART** |  |  |
| Proportion starting ART at CD4 cell count of 350 | 75% | Assumed |
| Annual ART entry rate if CD4 cell count >350 | 0.05 | Assumed |
| Reduction in sexual infectivity due to ART, % | 90% | ([18](#_ENREF_18)) |
| Reduction in injection infectivity due to ART, % | 50% | ([19](#_ENREF_19)) |
| **Progression Rates** |  |  |
| From asymptomatic to symptomatic | 0.152 | ([20](#_ENREF_20)) |
| From symptomatic to AIDS |  |  |
| Untreated | 0.303 | ([20](#_ENREF_20)) |
| Treated | 0.165 | ([20](#_ENREF_20)) |
| ^a^This number was calculated to balance total contacts amongst women with total sexual contacts amongst all men. | | |

1. WHO. Mortality Country Fact Sheet 2006 - Japan. Geneva: 2006.

2. Palella FJ, Deloria-Knoll M, Chmiel JS, Moorman AC, Wood KC, Greenberg AE, et al. Survival Benefit of Initiating Antiretroviral Therapy in HIV-Infected Persons in Different CD4+ Cell Strata. Ann Intern Med. 2003;138(8):620-6.

3. Ickovics JR, Hamburger ME, Vlahov D, Schoenbaum EE, Schuman P, Boland RJ, et al. Mortality, CD4 Cell Count Decline, and Depressive Symptoms Among HIV-Seropositive Women. JAMA: The Journal of the American Medical Association. 2001;285(11):1466-74.

4. Dunn D, Woodburn P, Duong T, Peto J, Phillips A, Gibb D, et al. Current CD4 Cell Count and the Short-Term Risk of AIDS and Death before the Availability of Effective Antiretroviral Therapy in HIV-Infected Children and Adults. Journal of Infectious Diseases. 2008;197(3):398-404.

5. Koerner J, Shiono S, Kaneko N, Shingae A, Ichikawa S, editors. Survey investigating homosexual behavior and attraction among adult males used to estimate HIV/AIDS prevalence and incidence among MSM in Japan. Japan-German AIDS Symposium; 2010 9-12 May 2010; Tokyo.

6. Network APaI. Classification of Reported HIV/AIDS Infections in 2010. 2011.

7. Quinn TC, Wawer MJ, Sewankambo N, Serwadda D, Li C, Wabwire-Mangen F, et al. Viral load and heterosexual transmission of human immunodeficiency virus type 1. Rakai Project Study Group. N Engl J Med. 2000;342(13):921-9. Epub 2000/03/30.

8. Mastro TD, de Vincenzi I. Probabilities of sexual HIV-1 transmission. AIDS. 1996;10 Suppl A:S75-82. Epub 1996/01/01.

9. Hidaka Y, Ichikawa S, Koyano J, Urao M, Yasuo T, Kimura H, et al. Substance use and sexual behaviours of Japanese men who have sex with men: A nationwide internet survey conducted in Japan. BMC Public Health. 2006;6(1):239.

10. Munakata T, Tajima K. Japanese risk behaviors and their HIV/AIDS-preventive behaviors. AIDS Educ Prev. 1996;8(2):115-33. Epub 1996/04/01.

11. Kaneko N, Utsumi M, Ichikawa S. HIV Testing Behavior and HIV Preventive Behavior among Gay and Bisexual Men in Tokai Area. Journal of the Japan Society of Nursing Research. 2007;30(4):37-43.

12. Ichikawa S. Research Into HIV Prevention Methods and Interventions Amongst MSM in Japan. In: Ichikawa S, editor. Combined Research Reports of the MSM Research Group, 2008-2010. Nagoya: Nagoya City University; 2010.

13. Ono-Kihara M, Sato T, Kato H, Suguimoto-Watanabe S, Zamani S, Kihara M. Demographic and behavioral characteristics of non-sex worker females attending sexually transmitted disease clinics in Japan: a nationwide case-control study. BMC Public Health. 2010;10(1):106.

14. Davis KR, Weller SC. The effectiveness of condoms in reducing heterosexual transmission of HIV. Fam Plann Perspect. 1999;31(6):272-9. Epub 1999/12/30.

15. Bureau PaFS. HIV Antibody Testing Amongst Blood Donors in Japan. 2011.

16. Bureau PaFS. Antibody Testing at Public Health Centres in Japan. 2011.

17. McCusker J, Stoddard AM, Mayer KH, Zapka J, Morrison C, Saltzman SP. Effects of HIV antibody test knowledge on subsequent sexual behaviors in a cohort of homosexually active men. Am J Public Health. 1988;78(4):462-7.

18. Donnell D, Baeten JM, Kiarie J, Thomas KK, Stevens W, Cohen CR, et al. Heterosexual HIV-1 transmission after initiation of antiretroviral therapy: a prospective cohort analysis. The Lancet. 2010;375(9731):2092-8.

19. Long EF, Brandeau ML, Owens DK. The cost-effectiveness and population outcomes of expanded HIV screening and antiretroviral treatment in the United States. Ann Intern Med. 2010;153(12):778-89. Epub 2010/12/22.

20. Granich RM, Gilks CF, Dye C, De Cock KM, Williams BG. Universal voluntary HIV testing with immediate antiretroviral therapy as a strategy for elimination of HIV transmission: a mathematical model. The Lancet. 2009;373(9657):48-57.
