# Supplementary material for: Projecting HIV Transmission in Japan
Source: PLoS One. 2012 Aug 20;7(8):e43473. doi: 10.1371/journal.pone.0043473 (PMC3423344; doi:10.1371/journal.pone.0043473)
Supplement: Table S3 — Initial values for the populations of risk groups. (DOCX) [file pone.0043473.s004.docx]

|  | **X1** | **X2** | **X3** | **X4** | **X5** | **X6** | **X7** | **X8** | **X9** | **X10** |
| --- | --- | --- | --- | --- | --- | --- | --- | --- | --- | --- |
| MSM | 422,466 | 248,115 | 4035 | 2370 | 1281 | 188 | 564 | 0 | 0 | 981 |
| Male | 23,541,451 | 13,825,931 | 3281 | 1927 | 1042 | 153 | 459 | 0 | 0 | 2190 |
| Female | 23,616,306 | 13,869,894 | 2237 | 1314 | 710 | 104 | 313 | 0 | 0 | 473 |
