# Supplementary material for: Projecting HIV Transmission in Japan
Source: PLoS One. 2012 Aug 20;7(8):e43473. doi: 10.1371/journal.pone.0043473 (PMC3423344; doi:10.1371/journal.pone.0043473)
Supplement: Table S4 — Modes of HIV transmission between risk groups. (DOCX) [file pone.0043473.s005.docx]

|  | **MSM** | **Male** | **Female** |
| --- | --- | --- | --- |
| **MSM** | Homosexual |  | Heterosexual |
| **Male** |  |  | Heterosexual |
| **Female** | Heterosexual | Heterosexual |  |
