# Supplementary material for: Projecting HIV Transmission in Japan
Source: PLoS One. 2012 Aug 20;7(8):e43473. doi: 10.1371/journal.pone.0043473 (PMC3423344; doi:10.1371/journal.pone.0043473)
Supplement: Table S5 — Parameters used in sensitivity analysis. (DOCX) [file pone.0043473.s006.docx]

| **Variable** | **Value** | **Possible Range** |
| --- | --- | --- |
| Number of MSM | 682,800 | 450,648 – 908,125 |
| Case-finding probability | 10% | 2% - 20% |
| Rate of ART Treatment for CD4 count= 350 mg/mL | 75% | 50-100% |
| Number of sexual partners per year |  |  |
| MSM | 5.5 | 3 – 7 |
| Low-risk men | 1.1 | 0.8 – 2 |
| Female partners of MSM | 0.1 | 0.01 – 0.3 |
| Low-risk women | 1.12* | Calculated^a^ |
| Condom use |  |  |
| MSM | 0.37 | 0.2 – 0.5 |
| Low-risk population | 0.2 | 0.1 – 0.3 |
| ^a^This value is calculated to balance with the total number of male sexual contacts with women. | | |
