# Supplementary material for: Projecting HIV Transmission in Japan
Source: PLoS One. 2012 Aug 20;7(8):e43473. doi: 10.1371/journal.pone.0043473 (PMC3423344; doi:10.1371/journal.pone.0043473)
Supplement: File S1 — Mathematical Model and Equations. (DOCX) [file pone.0043473.s007.docx]

**Supporting Information S1: Mathematical Model and Equations**

1. Epidemic Model Framework
   1. Main Model Structure

In order to estimate population-wide HIV transmission and progression rates, we developed a deterministic compartmental model, capturing transmission through both heterosexual and homosexual contacts.

Based on the current situation in the Japanese HIV epidemic, we subdivided our target population into three groups: men who have sex with men (MSM), low-risk men and low-risk women. Once infected, individuals progress though asymptomatic, symptomatic, and AIDS stages. The model separates the basic compartments for these disease stages by serostatus (identified/unidentified) and treatment status (treated/untreated) (Figure S1). The definition of all symbols can be found in Table S1, and initial values used for all parameters are shown in Table S2.

Based on the framework of our model, we developed differential equations for each risk group. The complete model comprises 30 equations. We coded these equations, initial values and parameters using MATLAB R2010a. We ran our program for 20 years. The 10 equations for each risk group are:

$$\frac{dX_{i,1}}{dt}=\rho_{1}^{i}\sum_{\forall j} X_{i,j}-\psi_{1}^{i}X_{i,1}+\omega_{2}^{i}X_{i,2}-\left(\sum_{j\geq3} \lambda_{i,j}(t)\right)X_{i,1}-(\mu_{1}^{i}+b_{1}^{i})X_{i,1}$$

$$\frac{dX_{i,2}}{dt}=\psi_{1}^{i}X_{i,1}-\omega_{2}^{i}X_{i,2}-\left(\sum_{j\geq3} \lambda_{i,j}(t)\right)X_{i,2}-(\mu_{2}^{i}+b_{2}^{i})X_{i,2}$$

$$\frac{dX_{i,3}}{dt}=\left(\sum_{j\geq3} \lambda_{i,j}(t)\right)X_{i,1}+\left(\sum_{j\geq3} \lambda_{i,j}(t)\right)X_{i,2}-(\psi_{3}^{i}+\nu_{3}^{i})X_{i,3}-(\theta_{3}^{i}+\mu_{3}^{i}+b_{3}^{i})X_{i,3}$$

$$\frac{dX_{i,4}}{dt}=(\psi_{3}^{i}+\nu_{3}^{i})X_{i,3}-(\theta_{4}^{i}+\mu_{4}^{i}+b_{4}^{i})X_{i,4}$$

$$\frac{dX_{i,5}}{dt}=\theta_{3}^{i}X_{i,3}-(\psi_{5}^{i}+\nu_{5}^{i})X_{i,5}-(\theta_{5}^{i}+\mu_{5}^{i}+b_{5}^{i})X_{i,5}$$

$$\frac{dX_{i,6}}{dt}=\theta_{4}^{i}(1-\phi_{4}^{i})X_{i,4}+(\psi_{5}^{i}+\nu_{5}^{i})(1-\phi_{5}^{i})X_{i,5}-(\theta_{6}^{i}+\alpha_{6}^{i}+\mu_{6}^{i}+b_{6}^{i})X_{i,6}$$

$$\frac{dX_{i,7}}{dt}=\theta_{4}^{i}\phi_{4}^{i}X_{i,4}+(\psi_{5}^{i}+\nu_{5}^{i})\phi_{5}^{i}X_{i,5}+\alpha_{6}^{i}X_{i,6}-(\theta_{7}^{i}+\mu_{7}^{i}+b_{7}^{i})X_{i,7}$$

$$\frac{dX_{i,8}}{dt}=\theta_{5}^{i}X_{i,5}-(\psi_{8}^{i}+\nu_{8}^{i})X_{i,8}-(\mu_{8}^{i}+b_{8}^{i})X_{i,8}$$

$$\frac{dX_{i,9}}{dt}=\theta_{6}^{i}X_{i,6}+(\psi_{8}^{i}+\nu_{8}^{i})(1-\phi_{8}^{i})X_{i,8}-\alpha_{9}^{i}X_{i,9}-(\mu_{9}^{i}+b_{9}^{i})X_{i,9}$$

$$\frac{dX_{i,10}}{dt}=\theta_{7}^{i}X_{i,7}+(\psi_{8}^{i}+\nu_{8}^{i})\phi_{8}^{i}X_{i,8}+\alpha_{9}^{i}X_{i,9}-(\mu_{10}^{i}+b_{10}^{i})X_{i,10}$$

where i correspond to 1: MSM, 2: male, 3: female

- 1. Target Population

Our target population is adults aged 15-59 years old. The rate at which people enter the target group is composed from the background population growth rate and the speed of reaching adulthood. In the same way, the maturation rate is the sum of the background mortality rate and the rate of ageing.

HIV prevalence in risk group i:

$$\text{p}_{\text{i}}=\frac{\text{Number of People living with HIV in risk group i}}{\text{Population of risk group i}}$$

Entry Rates:

$$\rho\text{=}-\ln(1-\frac{\text{15 years old population}}{\text{ 15-59 years old population}})+\text{growth rate}$$

Maturation Rates:

$$\mu\text{=}-\ln(1-\frac{\text{59 years old population}}{\text{ 15-59 years old population}})+\text{mortality rate}$$

Initial values for the population of each group are shown in Table S3, calculated from population size and prevalence information in existing studies.

- 1. Transmission Forces

Susceptible individuals can become infected in two ways: heterosexual and homosexual contact. Table S4 shows the details of potential modes of infection between any two risk groups. We consider heterosexual transmission to be possible for MSM as well.

- - 1. Common transmission formulae

The probability that men are not infected by HIV-positive women in compartment j, though one heterosexual contact, $N_{-}M_{j}$ ( *j*=3~10) is：

$$N_{-}M_{3}=\left[1-\left(\frac{X_{3,3}n_{3}^{O}(1-u_{3}^{O}\kappa)}{CT_{F}}\sigma_{f,m}^{a}\right)\right];N_{-}M_{4}=\left[1-\left(\frac{X_{3,4}n_{3}^{O}(1-r_{1})(1-u_{3}^{O}\kappa)}{CT_{F}}\sigma_{f,m}^{a}\right)\right]$$

$$N_{-}M_{5}=\left[1-\left(\frac{X_{3,5}n_{3}^{O}(1-u_{3}^{O}\kappa)}{CT_{F}}\sigma_{f,m}^{S}\right)\right];N_{-}M_{6}=\left[1-\left(\frac{X_{3,6}n_{3}^{O}(1-r_{1})(1-u_{3}^{O}\kappa)}{CT_{F}}\sigma_{f,m}^{S}\right)\right]$$

$$N_{-}M_{7}=\left[1-\left(\frac{X_{3,7}n_{3}^{O}(1-r_{1})(1-u_{3}^{O}\kappa)}{CT_{F}}\sigma_{f,m}^{S}(1-r_{2})\right)\right];N_{-}M_{8}=\left[1-\left(\frac{X_{3,8}n_{3}^{O}(1-u_{3}^{O}\kappa)}{CT_{F}}\sigma_{f,m}^{AIDS}\right)\right]$$

$N_{-}M_{9}=\left[1-\left(\frac{X_{3,9}n_{3}^{O}(1-r_{1}')(1-u_{3}^{O}\kappa)}{CT_{F}}\sigma_{f,m}^{AIDS}\right)\right];N_{-}M_{10}=\left[1-\left(\frac{X_{3,10}n_{3}^{O}(1-r_{1}')(1-u_{3}^{O}\kappa)}{CT_{F}}\sigma_{f,m}^{AIDS}(1-r_{2})\right)\right]$ where $CT_{F}$is the total heterosexual contacts among women:

$$CT_{F}=(\sum_{j=1,2,3,5,8} X_{3,j})n_{3}^{O}(1-u_{3}^{O}\kappa)+(\sum_{j=4,6,7} X_{3,j})n_{3}^{O}(1-r_{1})(1-u_{3}^{O}\kappa)+(\sum_{j=9,10} X_{3,j})n_{3}^{O}(1-r_{1}')(1-u_{3}^{O}\kappa)$$

The probability that MSM are not infected by HIV-positive MSM in compartment j though one homosexual contact, $N_{-}MSM_{j}$ (*j*=3~10) is：

$N_{-}MSM_{3}=\left[1-\left(\frac{X_{1,3}n_{1}^{S}(1-u_{1}^{S}\kappa)}{CT_{MSM}}\sigma_{m,m}^{a}\right)\right];N_{-}MSM_{4}=\left[1-\left(\frac{X_{1,4}n_{1}^{S}(1-r_{1})(1-u_{1}^{S}\kappa)}{CT_{MSM}}\sigma_{m,m}^{a}\right)\right]$ $N_{-}MSM_{5}=\left[1-\left(\frac{X_{1,5}n_{1}^{S}(1-u_{1}^{S}\kappa)}{CT_{MSM}}\sigma_{m,m}^{S}\right)\right];N_{-}MSM_{6}=\left[1-\left(\frac{X_{1,6}n_{1}^{S}(1-r_{1})(1-u_{1}^{S}\kappa)}{CT_{F}}\sigma_{m,m}^{S}\right)\right]$ $N_{-}MSM_{7}=\left[1-\left(\frac{X_{1,7}n_{1}^{S}(1-r_{1})(1-u_{1}^{S}\kappa)}{CT_{MSM}}\sigma_{m,m}^{S}(1-r_{2})\right)\right];N_{-}MSM_{8}=\left[1-\left(\frac{X_{1,8}n_{1}^{S}(1-u_{1}^{S}\kappa)}{CT_{MSM}}\sigma_{m,m}^{AIDS}\right)\right]$ $N_{-}MSM_{9}=\left[1-\left(\frac{X_{1,9}n_{1}^{S}(1-r_{1}')(1-u_{1}^{S}\kappa)}{CT_{MSM}}\sigma_{m,m}^{AIDS}\right)\right];N_{-}MSM_{10}=\left[1-\left(\frac{X_{1,10}n_{1}^{S}(1-r_{1}')(1-u_{1}^{S}\kappa)}{CT_{MSM}}\sigma_{m,m}^{AIDS}(1-r_{2})\right)\right]$ where $CT_{MSM}$ is the total number of homosexual contacts.

$$CT_{MSM}=(\sum_{j=1,2,3,5,8} X_{1,j})n_{1}^{S}(1-u_{1}^{S}\kappa)+(\sum_{j=4,6,7} X_{1,j})n_{1}^{S}(1-r_{1})(1-u_{1}^{S}\kappa)+(\sum_{j=9,10} X_{1,j})n_{1}^{S}(1-r_{1}')(1-u_{1}^{S}\kappa)$$

$N_{-}F_{i,j}$(i=1,2; *j*=3~10) is the probability that women are not infected by HIV-positive men in risk group i, and compartment j, though one heterosexual contact.

$$N_{-}F_{i,3}=\left[1-\left(\frac{X_{i,3}n_{i}^{O}(1-u_{i}^{O}\kappa)}{CT_{M}}\sigma_{m,f}^{a}\right)\right];N_{-}F_{i,4}=\left[1-\left(\frac{X_{i,4}n_{i}^{O}(1-r_{1})(1-u_{i}^{O}\kappa)}{CT_{M}}\sigma_{m,f}^{a}\right)\right]$$

$$N_{-}F_{i,5}=\left[1-\left(\frac{X_{i,5}n_{i}^{O}(1-u_{i}^{O}\kappa)}{CT_{M}}\sigma_{m,f}^{S}\right)\right];N_{-}F_{i,6}=\left[1-\left(\frac{X_{i,6}n_{i}^{O}(1-r_{1})(1-u_{i}^{O}\kappa)}{CT_{M}}\sigma_{m,f}^{S}\right)\right]$$

$$N_{-}F_{i,7}=\left[1-\left(\frac{X_{i,7}n_{i}^{O}(1-r_{1})(1-u_{i}^{O}\kappa)}{CT_{M}}\sigma_{m,f}^{S}(1-r_{2})\right)\right];N_{-}F_{i,8}=\left[1-\left(\frac{X_{i,8}n_{i}^{O}(1-u_{i}^{O}\kappa)}{CT_{M}}\sigma_{m,f}^{AIDS}\right)\right]$$

$N_{-}F_{i,9}=\left[1-\left(\frac{X_{i,9}n_{i}^{O}(1-r_{1}')(1-u_{i}^{O}\kappa)}{CT_{M}}\sigma_{m,f}^{AIDS}\right)\right];N_{-}F_{i,10}=\left[1-\left(\frac{X_{i,10}n_{i}^{O}(1-r_{1}')(1-u_{i}^{O}\kappa)}{CT_{M}}\sigma_{m,f}^{AIDS}(1-r_{2})\right)\right]$Where $CT_{M}$is the total number of heterosexual contacts for men.

$$CT_{M}=\sum_{i=1,2} \left[(\sum_{j=1,2,3,5,8} X_{i,j})n_{i}^{O}(1-u_{i}^{O}\kappa)+(\sum_{j=4,6,7} X_{i,j})n_{i}^{O}(1-r_{1})(1-u_{i}^{O}\kappa)+(\sum_{j=9,10} X_{i,j})n_{i}^{O}(1-r_{1}')(1-u_{i}^{O}\kappa)\right]$$

Again i corresponds to 1: MSM, 2: Male, 3: Female; and j corresponds to the 10 compartments reflecting HIV progression (1: unidentified uninfected, 2: Identified uninfected, 3:unidentified asymptomatic, 4: Identified asymptomatic, 5: unidentified symptomatic, 6: Identified symptomatic, 7: Identified symptomatic with ART, 8: unidentified AIDS, 9: Identified AIDS, 10: Identified AIDS with ART).

- - 1. Transmission rates for each group

Transmission forces$\sum_{j\geq3} \lambda_{i,j}(t)$ for the three risk groups are:

$$\sum_{j\geq3} \lambda_{1,j}(t)=\sum_{j\geq3} \left\{\left.1-N_{-}M_{j}^{n_{1}^{O}(1-u_{1}^{O}\kappa)}\right\}\right.+\sum_{j\geq3} \left\{\left.1-N_{-}MSM_{j}^{n_{1}^{S}(1-u_{1}^{S}\kappa)}\right\}\right.$$

$$\sum_{j\geq3} \lambda_{2,j}(t)=\sum_{j\geq3} \left\{\left.1-N_{-}M_{j}^{n_{2}^{O}(1-u_{2}^{O}\kappa)}\right\}\right.$$

$$\sum_{j\geq3} \lambda_{3,j}(t)=\sum_{i=1,2} \sum_{j\geq3} \left\{\left.1-N_{-}F_{i,j}^{n_{3}^{O}(1-u_{3}^{O}\kappa)}\right\}\right.$$

1. Model Outputs

In order to capture the HIV epidemiological trend prevalence, incidence, and cumulative incidence were calculated:

The number of susceptible individuals in risk-group *i,*

$$S_{i}=X_{i,1}(t)+X_{i,2}(t)$$

The number of PLWHA in risk-group *i,*

$$I_{i}=\sum_{\text{j=3}}^{10} X_{i,j}(t)\text{;}$$

HIV prevalence in risk-group *i,*

$$P_{i}=\frac{\sum_{\text{j=3}}^{10} X_{i,j}(t)}{\sum_{\forall j} X_{i,j}(t)}$$

New infections in risk-group *i*,

$$NI_{i}=\left(\sum_{j\geq3} \lambda_{i,j}(t)\right)\times\left(X_{i,1}(t)+X_{i,2}(t)\right)$$

Cumulative new infections in risk-group *i,*

$$CI_{i}=\int_{0}^{\text{t}} \left(\left(\sum_{j\geq3} \lambda_{i,j}(t)\right)\times\left(X_{i,1}(t)+X_{i,2}(t)\right)\right)dt$$

Parameters used in multivariate sensitivity analysis are shown in Table S5.
